# Supplementary material for: Comprehensive Annotation and Functional Exploration of MicroRNAs in Lettuce
Source: Front Plant Sci. 2021 Dec 24;12:781836. doi: 10.3389/fpls.2021.781836 (PMC8739914; doi:10.3389/fpls.2021.781836)
Supplement: Supplementary file 3 [file Data_Sheet_9.ZIP › GSM2230752.plot/Lsa-miR2275_Lsat_1_v5_gn_3_100900.1_1377_TPlot.pdf]

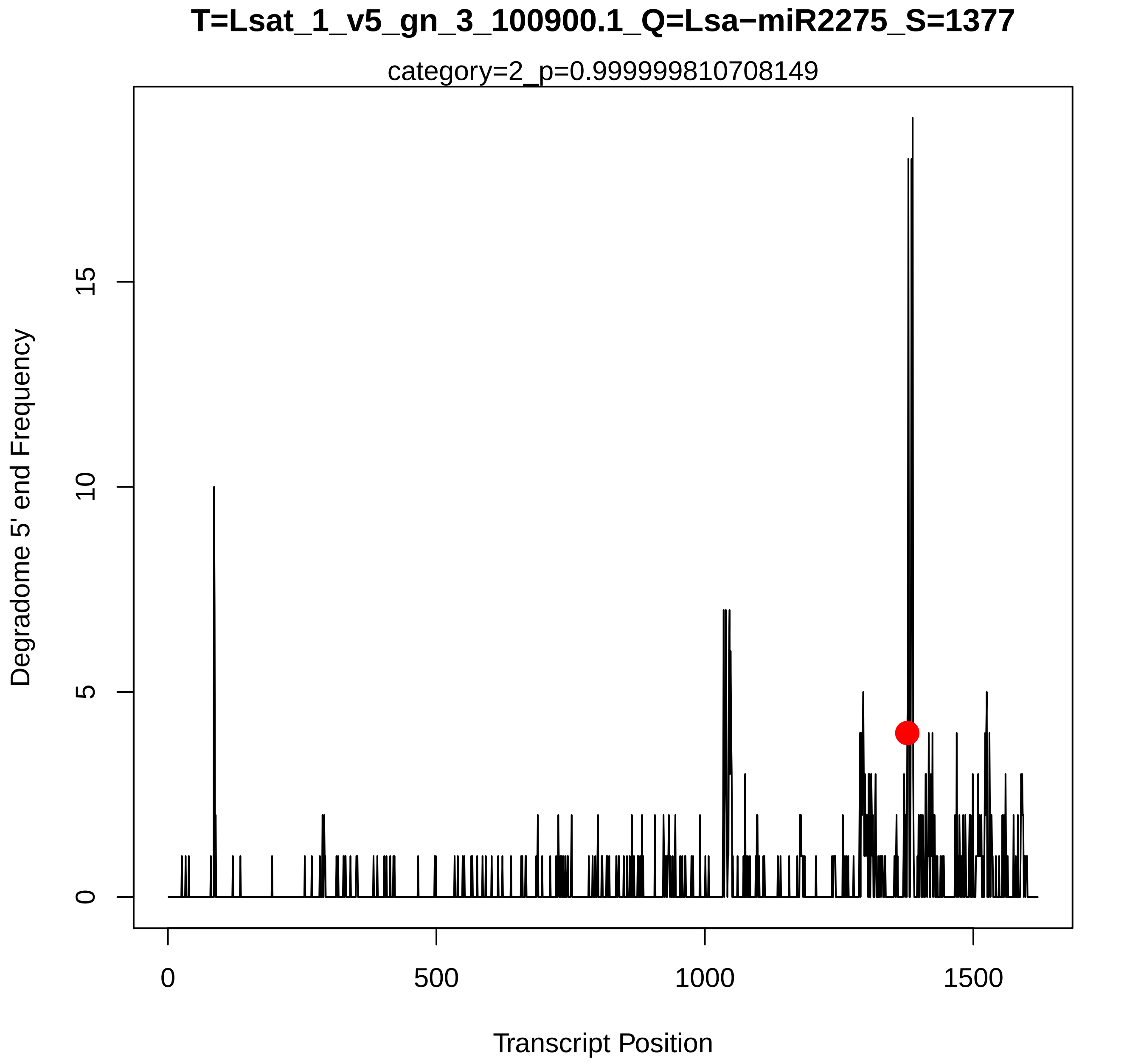
T=Lsat_1_v5_gn_3_100900.1_Q=Lsa−miR2275_S=1377
category=2_p=0.999999810708149
Degradome 5' end Frequency
Transcript Position
0
5
10
15
0
500
1000
1500
